# Epidemiology of *Plasmodium vivax* Malaria Infection in Nepal

**DOI:** 10.4269/ajtmh.18-0373

**Published:** 2018-07-16

**Authors:** Komal Raj Rijal, Bipin Adhikari, Prakash Ghimire, Megha Raj Banjara, Borimas Hanboonkunupakarn, Mallika Imwong, Kesinee Chotivanich, Kedar Prasad Ceintury, Bibek Kumar Lal, Garib Das Thakur, Nicholas P. J. Day, Nicholas J. White, Sasithon Pukrittayakamee

**Affiliations:** 1 Department of Clinical Tropical Medicine, Faculty of Tropical Medicine, Mahidol University, Bangkok, Thailand;; 2 Central Department of Microbiology, Tribhuvan University, Kirtipur, Kathmandu, Nepal;; 3 Mahidol-Oxford Tropical Medicine Research Unit (MORU), Faculty of Topical Medicine, Mahidol University, Bangkok, Thailand;; 4 Centre for Tropical Medicine and Global Health, Nuffield Department of Medicine, University of Oxford, Oxford, United Kingdom;; 5 Department of Molecular Tropical Medicine and Genetics, Faculty of Tropical Medicine, Mahidol University, Bangkok, Thailand;; 6 Epidemiology and Diseases Control Division (EDCD), Department of Health Service, Ministry of Health and Population, Teku, Kathmandu, Nepal;; 7 The Royal Institute, Grand Palace, Bangkok, Thailand

## Abstract

Malaria is endemic in the southern plain of Nepal which shares a porous border with India. More than 80% cases of malaria in Nepal are caused by *Plasmodium vivax*. The main objective of this study was to review the epidemiology of *P. vivax* malaria infections as recorded by the national malaria control program of Nepal between 1963 and 2016. National malaria data were retrieved from the National Malaria program in the Ministry of Health, Government of Nepal. The epidemiological trends and malariometric indicators were analyzed. Vivax malaria has predominated over falciparum malaria in the past 53 years, with *P. vivax* malaria comprising 70–95% of the annual malaria infections. In 1985, a malaria epidemic occurred with 42,321 cases (82% *P. vivax* and 17% *Plasmodium falciparum*). Nepal had experienced further outbreaks of malaria in 1991 and 2002. *Plasmodium falciparum* cases increased from 2005 to 2010 but since then declined. Analyzing the overall trend between 2002 (12,786 cases) until 2016 (1,009 cases) shows a case reduction by 92%. The proportion of imported malaria cases has increased from 18% of cases in 2001 to 50% in 2016. The current trends of malariometric indices indicate that Nepal is making a significant progress toward achieving the goal of malaria elimination by 2025. Most of the cases are caused by *P. vivax* with imported malaria comprising an increasing proportion of cases. The malaria control program in Nepal needs to counter importation of malaria at high risk areas with collaborative cross border malaria control activities.

## INTRODUCTION

Globally, an estimated 216 million cases of malaria were reported in 2016.^1^ Among them, 90% cases were in sub-Saharan Africa, 7% were in Asia, and 2% were reported from the Mediterranean region. In Southeast Asia, there has been a significant reduction (48% from 2010 compared with 2016) in malaria incidence.^1^ However, there could be discrepancies between national/subnational data and the World Malaria Report data, and so, adequate reporting of national data is critical.

A total of 1,009 confirmed malaria cases were reported in 2016 in Nepal. Microstratification of malaria risk in Nepal, in 2013, has categorized malaria risks into high, moderate, low, and no risk village development committees (VDCs)/municipalities. A total of 255 VDCs/municipalities are at risk of malaria (Supplemental Figure 1). Based on this microstratification, 47.9% of the total population live in the malaria-endemic region. Within the endemic region, 3.6% were from the high, 9.8% from moderate, and 34.5% from low malaria risk VDCs.^2^

Nepal’s National Health Program has identified malaria as a priority public health problem and is currently in the control/pre-elimination phase aiming to achieve elimination in 2025.^3^ With the availability of resources from the Global Fund to fight against AIDS, Tuberculosis, and Malaria, malaria control and elimination efforts have been strengthened since 2004.^4^ In 2004, Nepal switched from sulpfadoxine–pyrimethamine (SP) as first-line treatment of falciparum malaria to artemisinin-based combined therapies (ACTs) (artemether–lumefantrine) first in 13 high-risk districts and then in 2009, becoming the standard treatment regimen throughout the country.

Chloroquine and primaquine have been the first-line treatment of vivax malaria since 2009. Current malaria elimination activities take a multipronged approach: deployment of trained microscopists, use of rapid malaria diagnostic tests, deployment of ACTs, distribution of long-lasting insecticide-treated nets (LLINs), and the supply of Giemsa reagents for blood slide staining at the peripheral level health facilities where malaria is endemic.^5,6^

Nevertheless, there are several challenges for malaria control and elimination. Notably, the increasing overall importation of malaria from India.^6^ Together with importation, challenges associated with vivax malaria are the persistence of liver stages which give rise to relapse,^7–11^ the ability to survive at cooler temperatures,^8,12,13^ and the need for a diagnostic for glucose-6-phosphate dehydrogenase (G6PD) deficiency to provide radical cure safely with primaquine.^14,15^

Following the malaria elimination goal (targeted by 2025) adopted by the government of Nepal in 2011, in line with the “Global Malaria Strategic Plan,” specific and concerted strategies targeting the last remaining cases should be implemented. Most malaria control and elimination strategies focused on *Plasmodium falciparum* in Nepal, owing to the mortality and morbidity associated with it.^16,17^ In recent years, government reports indicate that more than 80% of the malaria cases are due to *Plasmodium vivax*.^6^ We analyzed the national data spanning over last 50 years (1963–2016) focusing on *P. vivax*. The main objective of this study was to explore the epidemiological trends of malaria, specifically, focusing on *P. vivax* in the current context of malaria elimination in Nepal.

## METHODS

### Study design.

This study is based on a retrospective analysis of malariometric data available from the Epidemiological Disease Control Division (EDCD), under the Ministry of Health and Population, Government of Nepal. Malaria data were available in a raw excel sheet as recorded by EDCD. The data presented in this study represent confirmed malaria cases diagnosed by microscopy. National malariometric indicators were analyzed for epidemiological trends. The data in this study represent the last 50 years (1963–2016). The study also analyzed malaria data to differentiate between indigenous and imported malaria cases in three highly malaria-endemic districts (Jhapa, Kailali, and Kanchanpur).

### Study sites.

Nepal is a landlocked country bordering with India on the south, east, and west and China on the north. Since the promulgation of new constitution in 2015, Nepal is divided into seven states and 77 districts with area of 147,181 km^2^.^18^ It occupies 0.3% of the land area of Asia and 0.03% of the land area of the world. Nepal is situated within latitudes 26°22′ N to 30°27′ N and longitude 80°4′ E to 88°12′ E. The topography of Nepal ranges between the lowland Terai at 70 M from sea level to the highest peak of the world, Mount Everest (8,848 M). Geographical divisions include Terai, hills, and mountains. The latest census in 2011 reported 26.5 million people in Nepal with a growth rate of 1.35 person per annum. More than 50% of the population lives in the Terai region of Nepal, where malaria is variably endemic.^19^

#### Jhapa district (eastern region).

Jhapa district is in the southeastern region of Nepal bordering with West Bengal state of India. The population of Jhapa district is 812,650 based on the national census in 2011.^19^ This district receives laborers returning home from the northeastern states of India where malaria is endemic. Residents of this district are from ethnic minorities that include Satar, Meche, and Tharu groups.

#### Kailali and Kanchanpur (far western region).

Kailali and Kanchanpur are adjoining districts in the far western region of Nepal on the southern plain that borders with Uttaranchal state of India. The total population of Kailali district is 775,709 and Kanchanpur district is 45,1248.^19^ The major population constitutes semi-urban residents with occupational exposure to malaria, specifically in relation to their visits to farms and jungles. These districts receive laborers returning home after working in Rajasthan, Delhi, Uttar Pradesh, Mumbai, Gujarat, and Orissa states of India and therefore are major hotspots for malaria importation. Ethnic minorities such as Rana Tharus and Dagaura Tharus are the main inhabitants of these districts.

### Data collection.

In Nepal, the passive malaria surveillance is carried out by different levels of health facilities such as health post, primary health center, and district hospital. Malaria cases recorded in the health facilities are reported monthly to the District Health Office (DHO)/District Public Health Office (DPHO). The data are reported to the Epidemiology and Diseases Control Division from DHO/DPHO through monthly Health Management Information System (HMIS) reports.^20^ Besides HMIS, an Early Warning Reporting System is used to record hospital-admitted cases and deaths due to malaria from the hospitals. As a part of malaria surveillance and control efforts, an electronic recording and reporting system referred to as Malaria Disease Information System has recently been implemented by EDCD. This includes an short message service alert system to central and district focal points once the cases are enrolled in health facilities and recorded.^6^

Malariometric indices were calculated based on the World Health Organization (WHO) definitions (Table 1).^20,21^ Furthermore, malaria data of two high-risk districts Kailali and Kanchanpur from the far western region and one moderate risk district Jhapa from the eastern region were collected directly from the DPHO. Field visits were made to ensure the compatibility of data that included cross-checking of reported data with the malaria register at local health facilities at study sites. The geographical mapping of the malaria cases based on the malariometric indicators was carried out using Arc GIS version 10.2 (Environmental Systems Research Institute [ESRI], Redlands, CA).

**Table 1:** Calculation of malariometric indices

Indices	Calculation
Annual blood examination rate =	$\frac{\text{Total number of slides examined}\times 100}{\text{Total population at risk of malaria}}$
Slide positivity rate =	$\frac{\text{Total number of positive slides}\times 100}{\text{Total slides examined}}$
Annual parasite index =	$\frac{\text{Total number of confirmed malaria cases}\times 1\text{,}000}{\text{Population at risk of malaria}}$
Slide *Plasmodium vivax* rate =	$\frac{\text{Total number of }P.\;\mathrm{vivax}\text{ positive slides}\times 100}{\text{Total slides examined}}$
Percentage *P. vivax* =	$\frac{\text{Total number of }P.\;\mathrm{vivax}\text{ cases}\times 100}{\text{Total malaria confirmed cases}}$

### Data analysis.

Data were first entered in Microsoft Excel 2010 and later analyzed. The trend of incidence of malaria and proportions of *P. vivax* cases in Nepal (1963–2016) were analyzed. The annual parasite incidence was calculated by using the total number of confirmed malaria cases as numerator and the number of population at risk of malaria as denominator and were expressed per 1,000 population at risk.

## RESULTS

### Annual trend of malaria incidence and proportion of *P. vivax* cases in 1963–2016.

**Figure 1.**
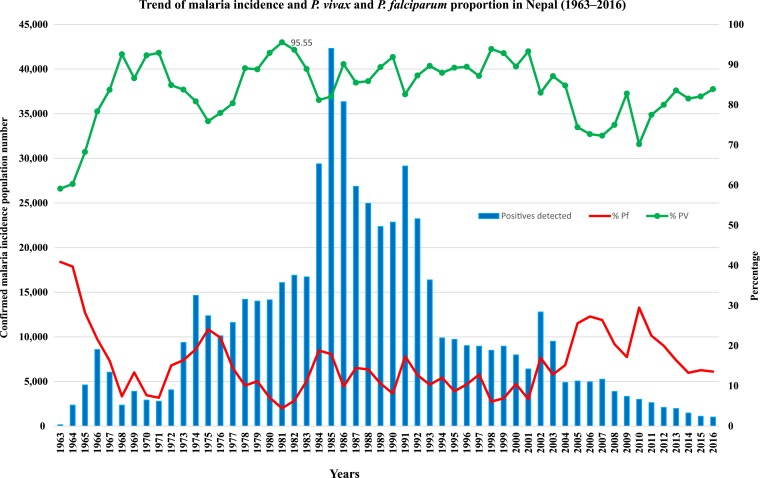
Yearly trend of malaria incidence and *Plasmodium vivax* proportion in Nepal (1963–2016). This figure appears in color at www.ajtmh.org.

The trend of malaria (either confirmed by microscopy or rapid diagnostic test) incidence and the proportion of *P. vivax* cases over the period of 1963–2016 were analyzed (Figure 1). The trends of malaria incidence are presented in the following paragraphs in 15- to 20-year periods.

### Period between 1963 and 1980.

In 1963, the total number of reported malaria cases was 159 (*P. vivax*: 94 and *P. falciparum*: 65), which rose to 14, 674 (*P. vivax*: 11, 842 and *P. falciparum*: 2, 805) by 1974. In 1963, *P. vivax* malaria comprised 60% (94/159) of diagnosed malaria cases. From 1967 (number of cases = 6,041) to 1974 (number of cases = 14, 674), there was a steady increase in *P. vivax* cases to comprise 80% of cases (11,842/14,647) by 1974. Between 1975 and 1980, the increase in *P. vivax* cases continued reaching 93% (13,147/14,148) in 1980.

**Figure 2.**
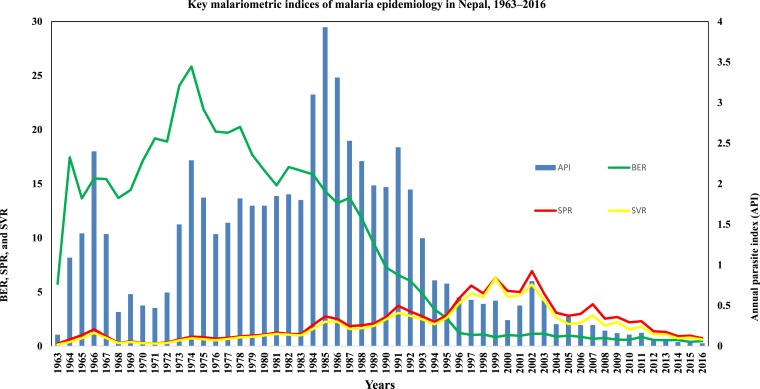
Changes in key malariometric indices of malaria epidemiology in Nepal (1963–2016). This figure appears in color at www.ajtmh.org.

In 1963, annual parasite index (API = total number of confirmed malaria cases × 1,000/population at risk of malaria) was reported to be 0.14 (159/1,174,324), which reached 2.29 (14,647/6,410,000) in 1974 with fluctuation over the years. It subsequently showed a downward trend falling to 1.73 (14,148/8,159,000) by 1980 (Figure 2).

### Period between 1981 and 2000.

During the 1980s, the proportion of vivax malaria (*P. vivax*: 95%; 15,834/16,902) steadily rose (highest point was 1981–1982), which then dropped before rising again. A huge outbreak occurred in 1985 with 42,321 cases, the highest number of reported malaria cases in Nepalese history (82% *P. vivax* and 17% *P. falciparum*). There was another malaria epidemic in 1991 and a total of 29, 135 (82% *P. vivax* and 17% *P. falciparum*) cases were reported. The total *P. vivax* proportion was more than 85% (20,280/23,234) in all years after 1992 until 2000s.

In 1981, API was reported to be 1.8 (16,087/8,682,000) and rose suddenly to 3.9 (42,321/10,758,000) in 1985. Since 1987, the API decreased less than three (26,866/10,605,651) and dipped to 1.96 (22,856/11,643,741) in 1990. The API showed a decreasing trend since 1994 and remained less than one (9,884/12,750,286) (Figure 2).

### Period between 2001 and 2016.

In 2002, the total reported malaria cases were 12,786, of which 83% (10,621/12,786) were *P. vivax* and 17% (2,165/12,786) *P. falciparum* (Figure 1). Although there was an overall decrease in the total number of malaria cases, the number of *P. falciparum* cases rose during 2005 till 2010. The proportion of *P. vivax* cases remained around 70–77% (in 2010, the proportion of *P. vivax* was 70%; 2,109/3,004). In between 2012 and 2016, *P. vivax* cases remained almost stable at more than 80% (in 2016, the proportion of *P. vivax* was 84%; 846/1,009). Overall there was a 92% reduction in malaria cases (12,786 cases in 2002 and 1,009 cases in 2016).

The API has dropped from 0.5 (6,408/13,215,972) in 2001 to 0.035 (1,009/28,621,688) in 2016 (Figure 2). The annual blood examination rate, slide positivity rate, and slide *P. vivax* rate also showed significant reductions during 1963–2016 (Figure 2).

### Importation of *P. vivax*.

**Figure 3.**
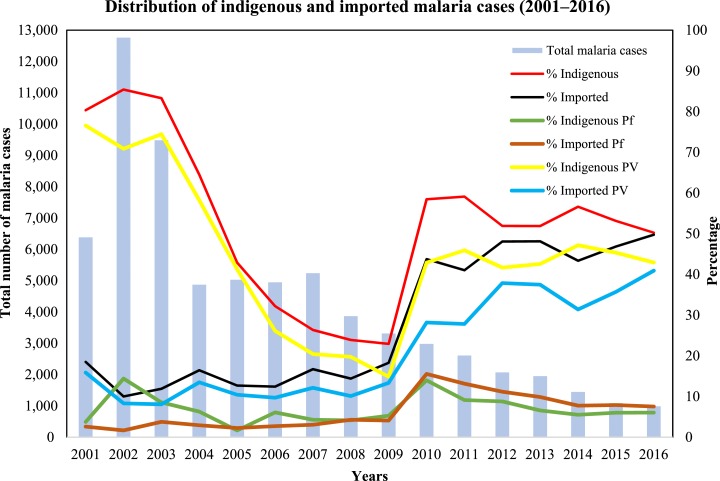
Distribution of indigenous and imported malaria cases (2001–2016). This figure appears in color at www.ajtmh.org.

A steady increase in imported malaria cases (mostly *P. vivax*) has been observed from 2001 onward. In 2001, only 18% (1,185/6,408) cases of malaria were imported, which rose to 50% (502/1,009) by 2016 (Figure 3). In 2011, a total of 2,631 malaria cases were reported. By 2016, there was 61.7% (1,622/2,631) reduction in malaria cases compared with 2011. Males were more likely to be infected by malaria than females (male, female ratio of 7:3). In 2016, of the 502 cases, 413 (92%) were caused by *P. vivax* and 76 (8%) were caused by *P. falciparum* and 13 (2.5%) cases were caused by both *P. vivax* and *P. falciparum* (Table 2).

**Table 2:** Distribution of malaria cases during 2012–2016 in Nepal

Characteristics	2012		2013		2014		2015		2016*	
	Number	%	Number	%	Number	%	Number	%	Number	%
Total *Plasmodium vivax* cases	1,664	79.1	1,649	83.5	1,154	78.6	901	81	846	83.8
Total *Plasmodium falciparum* cases	418	19.8	325	16.5	195	13.3	155	13.9	137	13.6
Total *Plasmodium* mix cases	22	1.1	–	–	120	8.1	56	5.1	26	2.6
Indigenous *P. vivax*	872	41.4	840	42.6	690	46.9	504	45.3	433	42.9
Imported *P. vivax*	792	37.6	740	37.5	461	31.4	397	35.7	413	40.9
Indigenous *P. falciparum*	184	8.7	130	6.5	81	5.5	67	6	61	6
Imported *P. falciparum*	234	11.1	195	9.8	114	7.8	88	7.9	76	7.6
Indigenous *P.* mix	10	0.4	–	–	58	3.9	20	1.8	13	1.3
Imported *P.* mix	12	0.6	–	–	62	4.2	36	3.2	13	1.3
Female	558	26.5	470	23.8	380	25.9	326	29.3	228	22.6
Male	1,546	73.5	1,504	76.2	1,089	74.1	786	70.7	781	77.4
Total malaria cases	2,104	–	1,974	–	1,469	–	1,112	–	1,009	–

* In 2016, total imported cases were 502 and total indigenous cases were 507.

### Malaria in three endemic districts.

The indigenous and imported cases of malaria in Nepal showed heterogeneous distribution in 2015. Indigenous cases were mostly concentrated in Kailali and Kanchanpur which are in the far western region of Nepal. Imported cases were distributed from the east to the west with few focal districts in the south, bordering with India, which suggests importation of malaria cases from India (Supplemental Figure 2). The distribution of malaria cases during 2013–2015 in two high-risk districts (Kailali and Kanchanpur) and one moderate risk district (Jhapa) showed that the number of indigenous malaria cases was significantly reduced. In Jhapa district, *P. falciparum* cases were decreasing. *Plasmodium vivax* cases were clustered among VDCs close to Indian border (Supplemental Figure 3). Also, in Kailali and Kanchanpur districts, clustering of *P. vivax* malaria cases was found in VDCs bordering with India (Supplemental Figures 4 and 5). For example, in Kailali district, Godawari VDC located in the lower altitude (Terai region) had more number of malaria cases compared with Kahirala VDC which is in the mountainous region (higher altitude). In addition, VDCs lying close to Indian border were found to have more cases of malaria compared with those which were farthest from the border.

## DISCUSSION

Until 1950s, 2 million cases of malaria were reported every year in Nepal with an estimated 10% mortality.^22^ Significant progress was then made in malaria control, with reduction of malaria cases and mortality after the malaria eradication program was launched in collaboration with the U.S. Agency for International Development in 1958 that comprised the widespread use of dichloro diphenyl trichloroethane (DDT) spray.^22,23^ Since 1963, *P. vivax* has predominated as the cause of malaria. The analysis of national epidemiological malaria data in Nepal shows a 10–20% fluctuation in the proportion of *P. vivax* incidence over the period of 1963–2016. Nevertheless, control and elimination of malaria pose significant challenge in Nepal because of the proportion of *P. vivax* cases imported from India in recent years.

### Period between 1963 and 1980.

During the period between 1963 and 1980, the proportion of infections caused by *P. falciparum* declined. This may be attributed to intense malaria control programs using DDT spray in forest fringes to destroy the indigenous anopheline vector.^24^ Consistent with the epidemiological pattern of Nepal, in India during 1960s, *P. vivax* was also responsible for 90% of the total malaria cases.^25,26^ Further evidence accrued to show that the main vector *Anopheles minimus* disappeared during the malaria eradication era.^4,24,27,28^

Globally, malaria cases increased by 231% between 1972 and 1976 with a 340% increment in Southeast Asia.^29^ Limited international support for malaria control program and worsening chloroquine and DDT resistance contributed to the increase in the disease burden.^30^ Realizing that eradication had failed, in 1978, Nepal adopted a new strategy following WHO’s change in policy from malaria eradication to control.^4,27^

### Period between 1981 and 2000.

There were two epidemics during this period, specifically in 1985 and 1991, mostly caused by *P. falciparum*. The epidemic of 1985 was attributed to an increase in chloroquine resistance in *P. falciparum*^4,27^ and vector resistance to DDT.^31^ Following the reports of resistance, the treatment regimen for falciparum malaria was changed from chloroquine to SP. In addition, synthetic pyrethroid insecticide was introduced as an indoor residual spraying agent replacing DDT and malathion.^4,27,32^

Another epidemic occurred in 1991, also in western and central Nepal; *P. vivax* remained at the same proportion (more than 80%) as in 1985. Various factors contributed to this epidemic including the massive population migration from mountainous regions to southern plains (especially mid-western and far western regions), heavy flooding in the resettlement areas, deforestation, and increased habitation around the foothills near densely forested areas. In response, there was massive use of synthetic pyrethroid insecticide lamda-cyhalothrin and α-cyhalothrin for indoor residual spraying in the endemic areas.^4,20,23,27^

### Period between 2001 and 2016.

There was one epidemic of *P. falciparum* in Kanchanpur district in 2002 in the far western region of Nepal, whereas *P. vivax* remained more than 80% of cases overall. This epidemic was linked to the resistance to SP.^17,23^ In a response to the epidemic, the national malaria control program adopted artemisinin combination therapy (artemether–lumefantrine) for the first time in 2004 in 13 priority districts. This was later expanded to other malaria-endemic districts (*N* = 28) in 2007. The treatment with ACT was rolled out nationwide in 2009.^33,34^

Between 2003 and 2010, *P. vivax* cases were in decreasing trend, which remained at 70% in 2010. During the same period, *P. falciparum* cases increased in eastern Nepal attributed to importation of cases from neighboring districts bordering with India.^23,25,35^ However, *P. vivax* regained upward trend in 2011 until 2016 and has remained greater than 80%. In recent years, increase in percentage of imported vivax malaria cases has been a main concern and is discussed in the following paragraph.

### Challenges of vivax malaria elimination (importation in Nepal).

Despite steady progress in achieving the goal of malaria elimination by 2025, *P. vivax* poses several challenges. One of the challenges of vivax malaria is the propensity to remain as a long-term reservoir for subclinical malaria infection.^7,25,36–38^ Radical treatment of vivax malaria targeting the persistent liver-stage hypnozoites requires use of primaquine. In Nepal, G6PD deficiency is concentrated in certain ethnic groups in endemic districts which varies between 1% and 10%, mostly among specific ethnic groups.^14^ Because primaquine causes hemolysis in individuals with G6PD deficiency, testing is required in at-risk populations before its administration.^14^ This is generally unavailable.

The increase in importation has changed malaria epidemiology in Nepal. There has been a decline in *P. falciparum* cases but persistence in *P. vivax* cases. Whereas this may be attributed to importation (mostly in population comprising labor migrants), it could also be due to different treatment regimens for two types of malaria (*P. vivax* and *P. falciparum*) in Nepal. At the peripheral health facilities, cases of malaria (without identifying the species) are likely to have been treated without primaquine. Three days chloroquine and 14 days primaquine was a standard treatment regimen for *P. vivax* malaria since 2009,^39^ whereas ACTs were used to treat falciparum cases since 2004.^34^

The importation of *P. vivax* malaria has been rising annually, specifically from 2010. In this study, high percentage of *P. vivax* malaria cases were imported compared with *P. falciparum*. Few studies in past have reported importation and the factors affecting it.^40^ Most of the imported cases had a travel history of being in the malaria-endemic region of India and were mostly male laborers.^35^

Nepal has more than 1,400 km of porous border with India. Previous studies have reported that high mobility of people for labor migration, particularly seasonal migration, who returned from malaria-endemic areas for *P. vivax* (Gujarat, Maharashtra, Assam, and West Bengal)^6,25^ contributes to the high importation of malaria to far western Nepal.^41,42^ Similar patterns of importation have been reported from other neighboring countries in Southeast Asia that include Bhutan, Sri Lanka, and Pakistan.^43–45^ Importation of malaria has been consistently reported from other Asian countries sharing borders such as China and Myanmar.^46^

### Malaria in three endemic districts.

Malaria high-risk districts (Kailali, Kanchanpur, and Jhapa) were mapped by malaria cases. An interesting finding consistent with all three districts were cases of *P. falciparum* that were few and scattered, whereas *P. vivax* were clustered among VDCs close to Indian border. High number of malaria cases in these districts could be due to high population movement between India and Nepal, where significant proportion of Nepalese youth travel for labor work and employment. Consistent with our findings, a spatiotemporal study conducted in Gujarat showed similar clustering of malaria cases (hot spots in regions) which were attributed to population movement.^47,48^ Risk maps in this study could be used for targeted malaria elimination efforts.^48^

### Recommended strategy.

Malaria control and elimination strategies are being expedited in Southeast Asia and the Greater Mekong region to stop the spread of artemisinin resistance from the region to westward.^49–52^ Multiple control measures, including mass drug administration for malaria hot spots, strengthening village malaria workers, and deployment of LLINs, are implemented with community engagement strategies wherein community members are trained and devolved with the responsibilities.^49,50^ The current malaria control program in Nepal may benefit by adopting community engagement strategies to intensify malaria control activities by collaborating with the communities in endemic districts.

### Strengths and limitations.

This is a first study analyzing the national malariometric data with a focus on *P. vivax* to guide the Nepal national malaria control program, particularly to target these cases which remain challenging because of its persistence and high burden of importation. This study could have benefitted by expanding the spatiotemporal analysis for additional targeting of clustered cases in different endemic regions. The data presented in this study represent confirmed malaria cases by microscopy. Malaria case reporting was supported by the trained human resources (e.g., malaria inspectors) allocated at the local level (malaria check posts) and routinely used the active case detection during eradication era in addition to the malaria surveillance system. Although there is a less difference on the confirmed malaria cases between national malaria data and the World Malaria Report by WHO, estimates could be discrepant because of the method used in projecting the number of cases such as using mathematical modeling. This study could not explore the detailed sociodemographics of the confirmed malaria cases because of the unavailability of these data. Future studies can benefit from detailed exploration of these characteristics in relation to importation of malaria focused on particular groups in endemic areas.

## CONCLUSION

The importation of malaria poses challenges to achieve malaria elimination goals by 2025. Most of the importation of cases occurred in the southern plain of Nepal bordering with India. The importation of *P. vivax* is one of the major challenges for both the current pre-elimination and elimination of malaria. The latent stage of this parasite and reservoir potential can cause persistence and relapse of malaria. Radical cure with primaquine needs to be deployed more widely. The malaria control program in Nepal can benefit by strengthening the health infrastructure (for active detection and treatment) with a focus on malaria-endemic districts with risk of high importation. In addition, active case detection by establishing malaria check posts in border areas with a joint cross border collaboration is urgently required to halt the current importation.

## Supplementary Material

Supplemental figures
