# Supplementary figures and images for: Epidemiology of Plasmodium vivax Malaria Infection in Nepal
Source: Am J Trop Med Hyg. 2018 Jul 16;99(3):680–7. doi: 10.4269/ajtmh.18-0373 (PMC6169153; doi:10.4269/ajtmh.18-0373)

# Situation analysis of malaria in Kanchanpur district

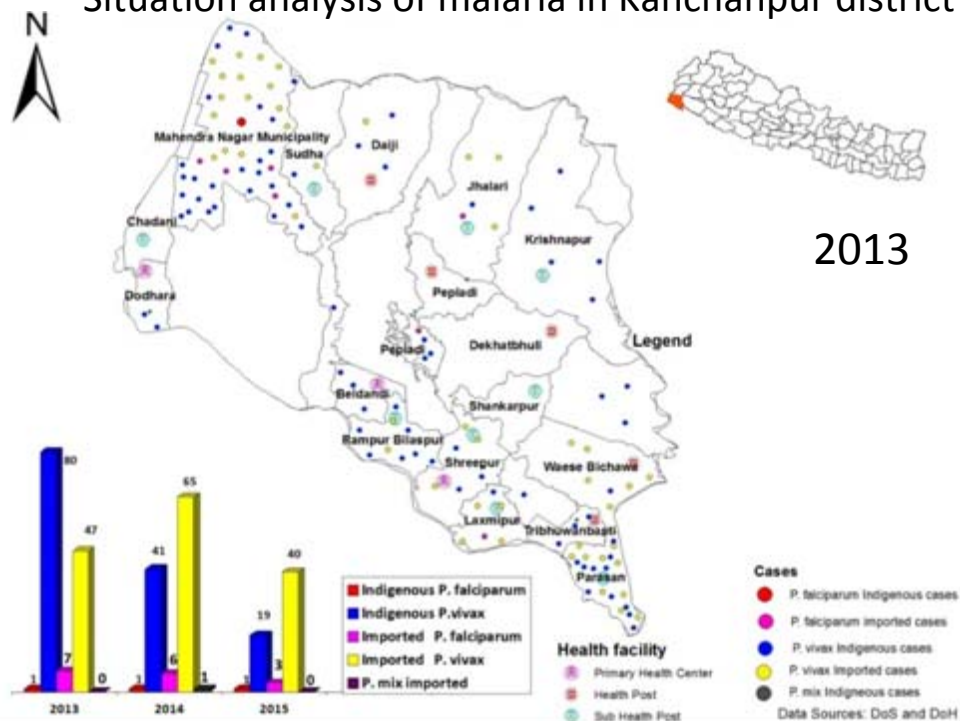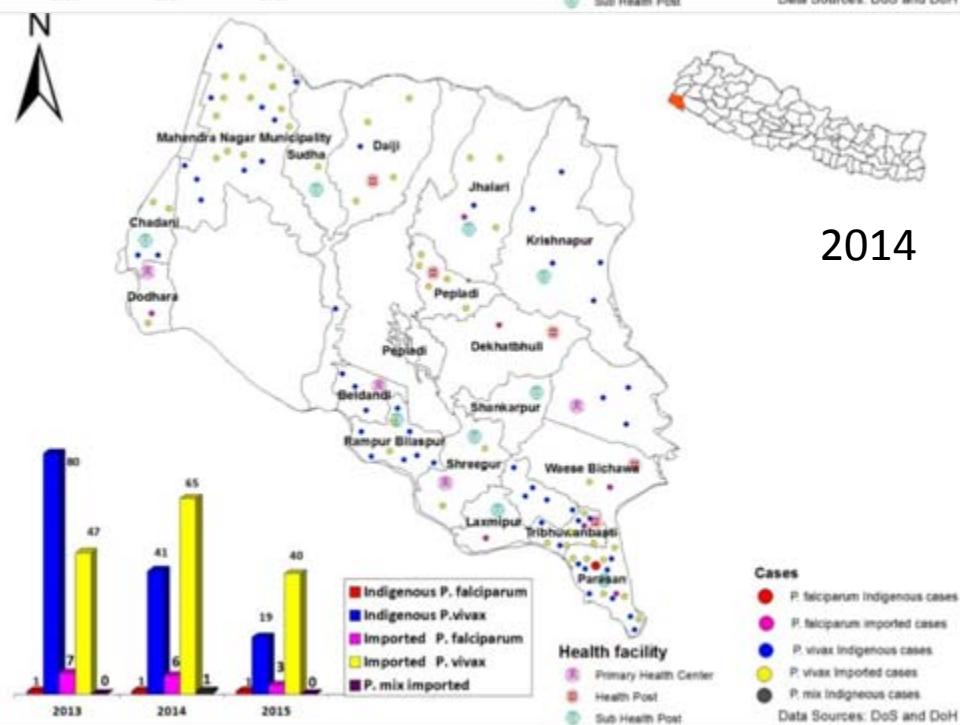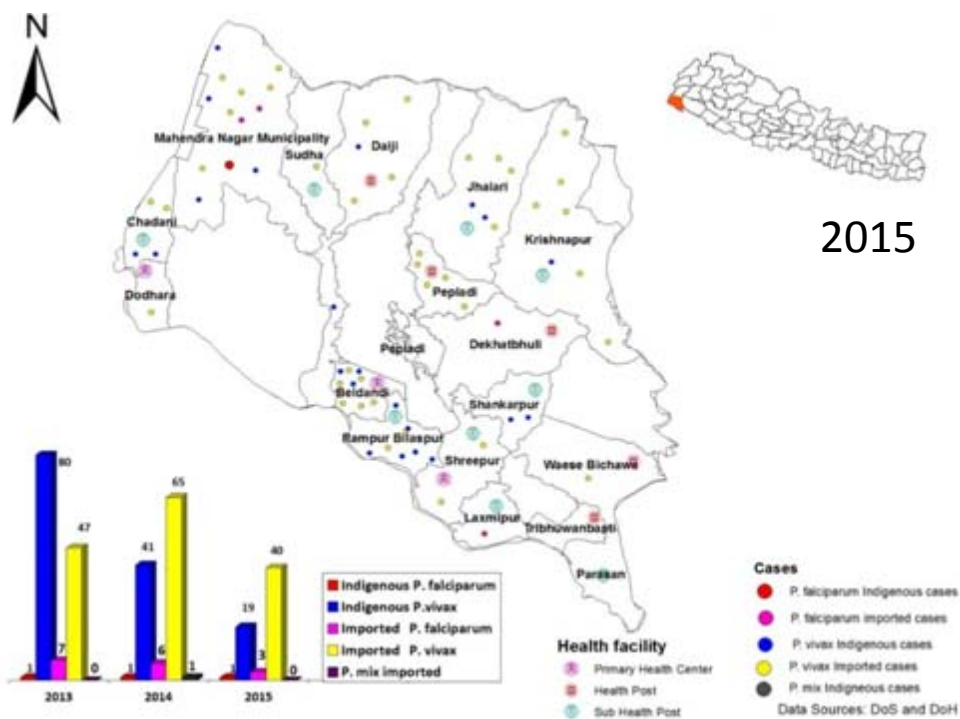

Supplement: Supplementary file 1 [file tpmd180373.SD1.pdf]

# Situation analysis of malaria in Kailali district

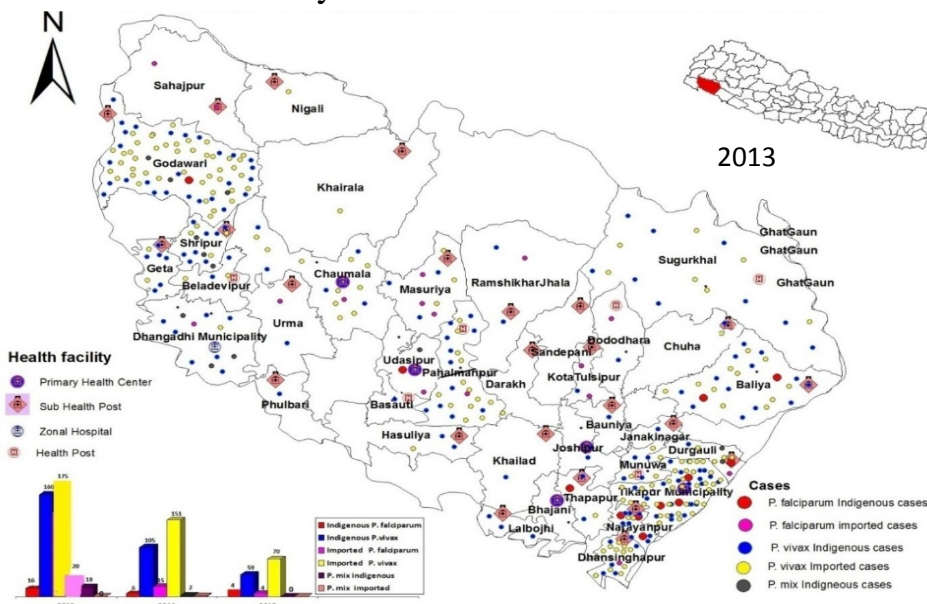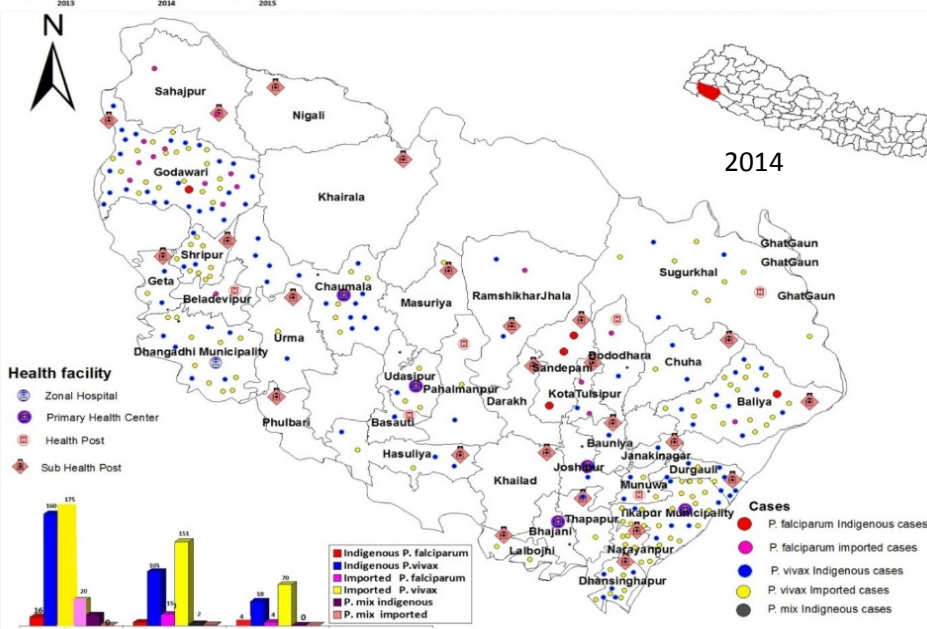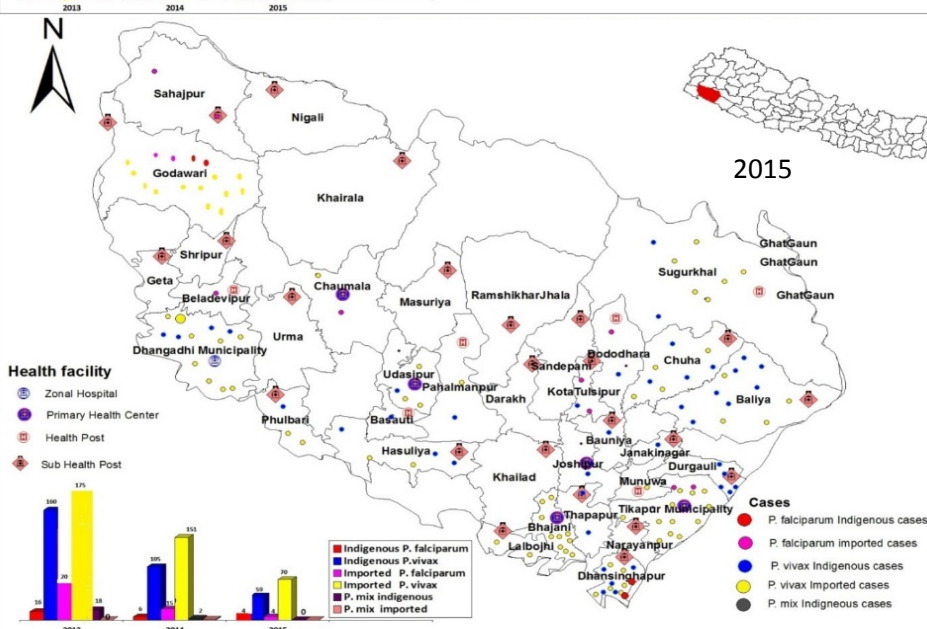

Supplement: Supplementary file 2 [file tpmd180373.SD2.pdf]

# Situation analysis of malaria in Jhapa district

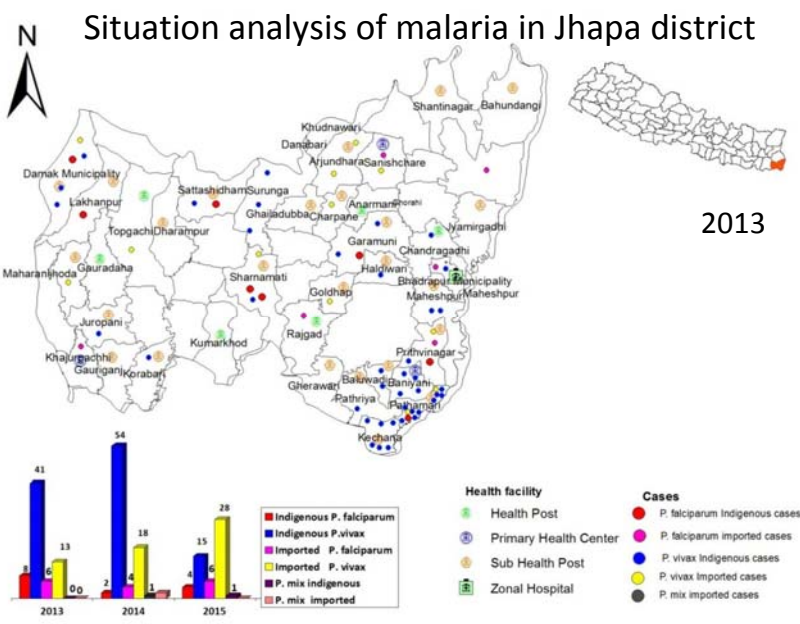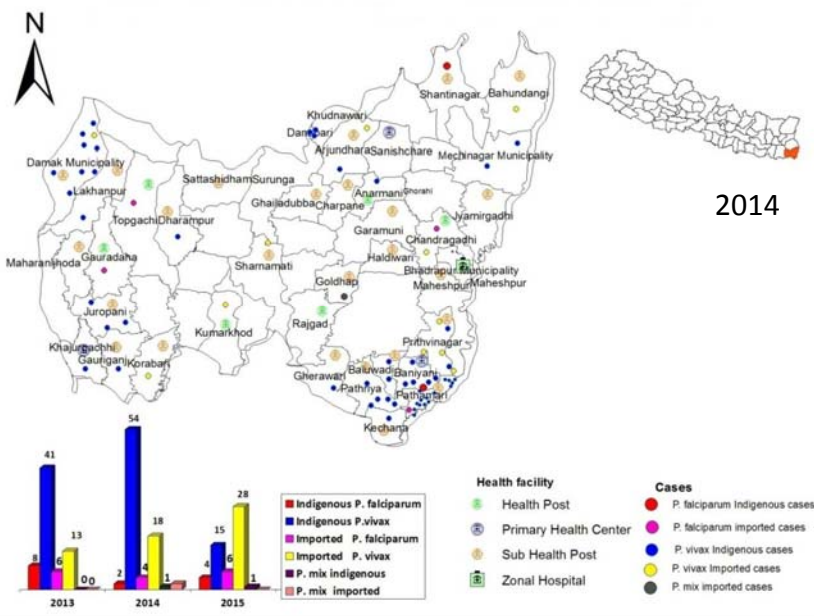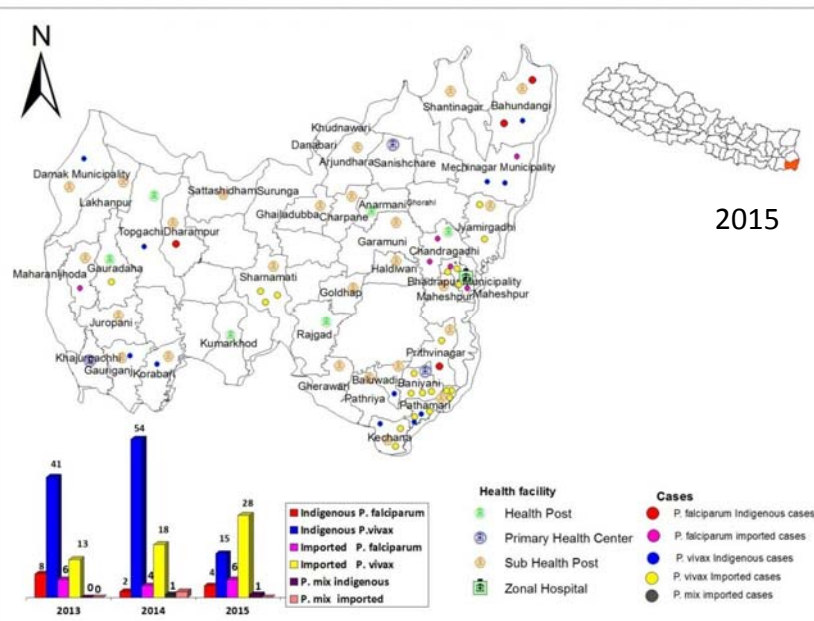

Supplement: Supplementary file 3 [file tpmd180373.SD3.pdf]

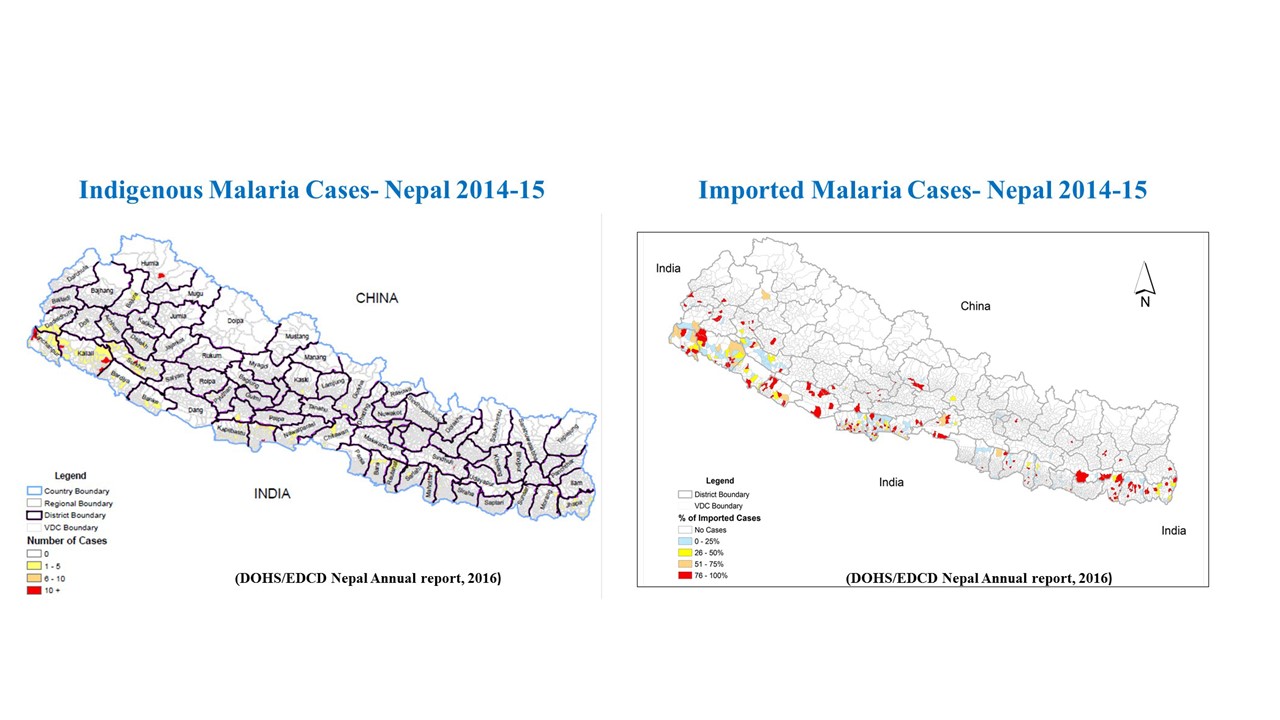

Supplement: Supplementary file 4 [file tpmd180373.SD4.jpg]

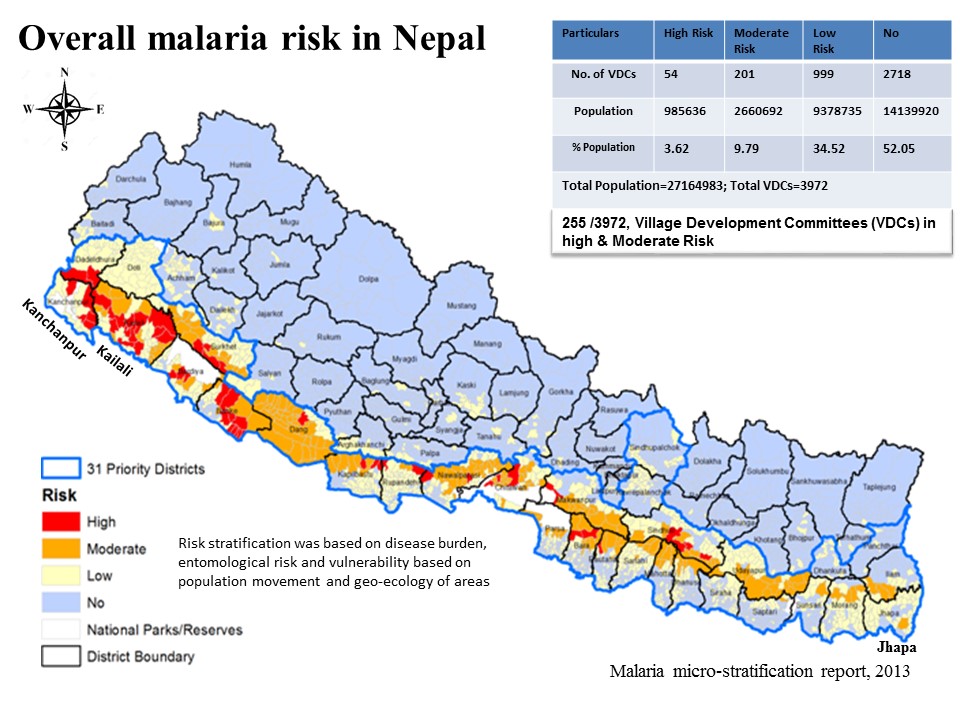

Supplement: Supplementary file 5 [file tpmd180373.SD5.jpg]
